# An overview: biomolecules from microalgae for animal feed and aquaculture

**DOI:** 10.1186/2241-5793-21-6

**Published:** 2014-05-19

**Authors:** Zahira Yaakob, Ehsan Ali, Afifi Zainal, Masita Mohamad, Mohd Sobri Takriff

**Affiliations:** Department of Chemical & Process Engineering Faculty of Engineering & Built Environment, University Kebangsaan Malaysia, 43600 Bangi, Malaysia; Centre for Energy Systems, National University of Sciences and Technology, Sector H-12, Islamabad, Pakistan

**Keywords:** Microalgae, Carotenoid, Biomolecules, Nutraceutical, Aquaculture, Animal feed

## Abstract

Despite being more popular for biofuel, microalgae have gained a lot of attention as a source of biomolecules and biomass for feed purposes. Algae farming can be established using land as well as sea and strategies can be designed in order to gain the products of specific interest in the optimal way. A general overview of the contributions of Algae to meet the requirements of nutrients in animal/aquaculture feed is presented in this study. In addition to its applications in animal/aquaculture feed, algae can produce a number of biomolecules including astaxanthin, lutein, beta-carotene, chlorophyll, phycobiliprotein, Polyunsaturated Fatty Acids (PUFAs), beta-1,3-glucan, and pharmaceutical and nutraceutical compounds which have been reviewed with respect to their commercial importance and current status. The review is further extended to highlight the adequate utilization of value added products in the feeds for livestock, poultry and aquaculture (with emphasis in shrimp farming).

## Introduction

Nutritional composition of algae is well documented and it mainly contains proteins, carbohydrates, lipids and trace nutrients, including vitamins, antioxidants, and trace elements. These all algal components have the characteristics to be a natural supplement in human and animal feed to replace synthetic components or meet the increasing demands of such ingredients. Increased consumption of animal proteins is creating a towering demand of high quality fish and animal feed to generate healthy proteins for humans. Fish meal is the protein source traditionally used in aquaculture diets but it is considered as limited and expensive. The nutritional contents of algae are rapidly gaining importance as a renewable source to substitute the conventional ingredients in the aquaculture/animal feed. Algae is defined as heterogeneous assemblage of organisms that range in size from tiny cells to giant seaweeds, thus algae mostly are photosynthetic species which includes eukaryotes and prokaryotic Cyanobacteria (blue-green algae) and live in aquatic habitats. Algae can be differentiated (by the body size and structure) either to microalgae (with algal bodies that need microscope to be observed) or to macroalgae (large enough to be seen with unaided eye) [1]. Algae culturing has been adopted since the late of 1800s and early 1900s, while microalgae mass culturing begin to develop since the achievement of Allen and Nelson in 1910 by cultivating *Chlorella* sp. for aquaculture purposes in Berlin, Germany [2]. To date, it seems that microalgae have the potential to play pivotal roles in order to remedy the energy, environment and food crisis prevailing in the world. This review encompasses the roles of algae in biomolecules production including astaxanthin, lutein, beta carotene, chlorophyll, phycobiliprotein, Polyunsaturated Fatty Acids (PUFAs), beta-1,3-glucan, and pharmaceutical and nutraceutical compounds. This review also emphasizes on the utilization of these biomolecules in livestock, poultry and aquaculture (and especially shrimp farming) feed.

## Review

### Production of value added products from algae

The global carotenoids market is projected to reach US$1.2 billion by the year 2015, because of the rising consumer awareness about health benefits offered by various carotenoids, and the shift towards healthy and natural food products (Global Industry Analysts, Inc http://www.strategyr.com/Carotenoids_Market_Report.asp/). According to a recently published report, global carotenoids market tends to reach US$1.3 billion by 2017 (http://www.prweb.com/pdfdownload/8849957.pdf). The nutraceuticals boom has also integrated carotenoid mainly on the claim of their proven antioxidant properties; carotenoids are usually considered important for industrial use in food products, cosmetics as vitamin supplements, health food products, and as feed additives for poultry, livestock, fish, and crustaceans [3]. In contrast, carotenoid in fish oil comes from microalgae, therefore it is logical to cultivate microalgae for carotenoids production [4]. Del Campo *et al*. [3] have reported that structure of more than 600 different carotenoids have been derived from a 40-carbon polyene chain. This paper will focus on carotenoids such as astaxanthin, lutein, beta-carotene and other high-valued molecules which are produced by microalgae (or in some case by macro algae) and Cyanobacteria and can be industrialized. Moreover, microalgae play a key role in high grade animal nutrition food from aquaculture to farm animals. Comprehensive nutritional and toxicological evaluations have demonstrated suitability of algal biomass as a valuable feed supplement or substitute for conventional animal feed sources [5]. Therefore, the usage of microalgae biomass for feed productions will be discussed in this review which might be helpful to encourage the trends of mass cultivation of microalgae for commercial and beneficial purposes.

### Astaxanthin

Astaxanthin, a carotenoid equipped with two asymmetric carbon located at the 3 and 3΄ position of the benzenoid rings on either end of the molecule, has been proposed as super vitamin E due to its natural antioxidant activities [6–8]. As a natural pigment, the major market for astaxanthin is as pigmentation source in aquaculture [9]. Natural astaxanthin can be produced by *Haematococcus* microalgae, *Chlorella zofingiensis*, *Chlorococcum* sp. and the yeast *Xanthophyllomyces dendrorhous*[10–13]. Studies regarding effects on lipid metabolism and antioxidant defense mechanisms revealed that astaxanthin is responsible for lowering the plasma lipid concentrations and enhancing antioxidant defense in apolipoprotein E knockout mice [14]. The increasing growth of salmonid farming has created an enormous demand for natural astaxanthin as pigment. Out of total carotenoid (astaxanthin, cantaxanthin), the astaxanthin was determined as the most important carotenoid in salmons and rainbow trouts [15]. Salmonid are unable to synthesize astaxanthin *de novo*, therefore carotenoid pigments must be supplied in their artificial aquaculture diet [16]. The carotenoid pigment astaxanthin has important applications and is routinely being used in the nutraceutical, cosmetics, food and feed industries [17]. In aquaculture feed, it has been used to culture salmon, shrimp, ornamental fish and sea bream. A recent report showed that *Haematococcus pluvialis* especially in dose of 3 g kg^-1^ feed administration may effectively enhance the antioxidant system and some biochemical parameters in rainbow trout [18]. Astaxanthin is not responsible only to provide pigmentation but also has been attributed with extraordinary potential for protecting the organism against a wide range of diseases, and has considerable potential and promising applications in human health [13]. Natural astaxanthin was also found effective to treat the patients with functional dyspepsia, caused by *Helicobacter pylori*[7]. UV radiation from sunlight is known as the most potent environmental risk factor in skin cancer pathogenesis, astaxanthin has been demonstrated as a protective tool against UVA-induced DNA alterations in human skin fibroblasts (1BR-3) [19].

Epidemiological studies have demonstrated a correlation between increased carotenoid intake and reduced incidence of coronary heart disease, certain cancer types, macular degeneration, and increased resistance to viral, bacterial, fungal and parasitic infection [20]. The price for astaxanthin varies in the market and depends on the percentage contents in algal source; however the overall maintained price of 5% astaxanthin is about US$1900 kg^-1^ (http://www.herbridge.com/bencandy.php?fid-55-id-23446-page-1.htm).

### Lutein

Lutein is another important carotenoid which can be found naturally in algae. It is prominent in human serum and foods [21]. Lutein has been used for the pigmentation of animal tissues and products, and it is considered important for the natural coloration of foods, drugs and cosmetics. It has been documented that it plays an active role in delaying chronic diseases, stimulating the immune response, hampering the development of cataracts and the progression of early atherosclerosis, and preventing blindness or decrease in vision caused by age-related macular degeneration [22–24]. Global lutein market in 2004 accounted for US$139 million and was considered as the fastest projected growth in individual carotenoids sales [3]. The recent market value of lutein is ~ US$233 million (2010), and is expected to reach US$309 million by 2018 with a compound annual growth rate of 3.6% (http://www.companiesandmarkets.com/Market/Food-and-Drink/Market-Research/The-Global-Market-for-Carotenoids/RPT988273). French marigold (*Tagetes patula*) is currently the most widely applied source for lutein production in the world. In the USA, two lutein containing products, Aztec Marigold and Tagetes have been commercialized. Lutein is usually produced from three different marigold flowers (*Tagetes patula* L.) (marigold orange, marigold yellow, and marigold red) but the marigold orange (MGO) variety contains the maximum amount of lutein [25]. However, mass plantation of marigold occupies a large land area and it is easily influenced by seasonal and climatic change [24]. Although an established commercial system for the production of lutein from microalgae does not exist yet, the basis for outdoor production of lutein-rich cells of strains of *Muriellopsis* sp. and *Scenedesmus* sp. at a pilot scale have already been set up [3]. Recently, there has been remarkable interest in dietary carotenoids with respect to their antioxidant properties and their ability to reduce the chances of some chronic diseases which occurred because of the free radicals. Possibly, carotenoids protect cells from oxidative stress by quenching singlet oxygen damage with a variety of mechanisms. Therefore, algae derived carotenoids could be a leading natural resource in the search of potential functional ingredients [26]. The microalgae biomass from chlorophycean is also considered as the major source of lutein, especially *Chlorella* sp., *Scenedesmus* sp. and *Muriellopsis* sp. [3, 24]. Laboratory studies showed that heterotrophically cultivated *Chlorella* sp. produce considerable amount of lutein [27]. The ability of heterotrophic growth in fermenters makes *Chlorella* sp. a potential alternative resource for commercial production of lutein [24]. Studies showed that more lutein will be produced by *Chlorella* sp. using Basal Medium compared to Kuhl Medium because of the high concentration of EDTA and minerals (500 mg l^-1^: 9.3 mg l^-1^) [22]. On the other hand, Wu *et al*. [24] have modeled lutein production by heterotrophic *Chlorella* sp. in batch and fed-batch cultures as a potential alternative source for commercial production.

### Beta-carotene

Beta-carotene is a pigment of increasing demand and a wide variety of market applications: as food coloring agent, pro-vitamin A (retinol) in food and animal feed, additive to cosmetics and multivitamin preparations, and as a health food product under the antioxidant category. The market value of beta-carotene was estimated about US$253 million in 2009 [3]. According to a report on the global market for carotenoids (September 2001), the market value of beta-carotene was estimated at around US$261 million in 2010 and this market is expected to grow to US$334 million by 2018 at a compound annual growth rate of 3% (http://www.companiesandmarkets.com/News/Chemicals/Beta-carotene-the-Largest-Carotenoid-Sector-is-Forecast-to-be-Worth-US-334-Mn-by-2018/NI2439).

Because of limited supply from natural sources, a number of companies have introduced synthetically produced carotenoids to meet the high demands in the global market. Beta-carotene, a provitamin A is the only carotenoid which has the potential to form two molecules of vitamin A (retinol) [28]. Natural production of beta-carotene can be produced by the microalgae genus *Dunaliella*, strains that have shown to change their color from green to orange may produce and accumulate large amount of cellular beta-carotene [29]. *Dunaliella* sp. occurs in oceans, brine lakes, salt marshes, salt lagoon and salt water ditches near sea, predominantly in water bodies containing more than 2 M salt and high level of magnesium. *Dunaliella* sp. is recognized as being the most halotolerant eukaryotic photosynthetic organism, showing a remarkable degree of adaptation to variety of salt concentrations from as low as 0.1 M to salt saturation about 4 M [30]. The unicellular halotolerant alga *Dunaliella bardawil* was shown to contain high concentration of beta-carotene, composed of the all-*trans* and the 9-*cis* isomers [28]. Beta-carotene is a structural analogue of vitamin A and its topical application can effectively be used to treat skin melasma [31]. It has been reported that a daily dose of *Dunaliella* sp. beta-carotene exerts a protective effect against exercise induced asthma probably through *in vivo* antioxidative effect [32]. Age-related macular degeneration is one of the most common eye diseases causing severe or permanent loss of vision, an antioxidant therapy using combinations of high dosage antioxidant vitamins C, E, beta-carotene, and zinc are employed [33, 34].

The price for natural beta-carotene depends on demand varying from US$300 to US$3000 kg^-1^. Purified beta-carotene is sold in vegetable oil form ranging from 1% to 20% of concentration depending on type of food products. Naturally produced purified beta-carotene is usually accompanied with other *Dunaliella* sp. carotenoid (carotenoid mix) which includes lutein, neoxanthin, zeaxanthin, violaxanthin, cryptoxanthin, and α-carotene. Variety of natural beta-carotene can be found for sale as health food and supplement under vitamin section. In powder form, natural beta-carotene is used for colorization and pro-vitamin A for animal (cattle and poultry) and aquaculture (shrimp and fish) feed [30].

### Chlorophyll

Chlorophyll is a green pigment and is an extremely important biomolecule, critical in photosynthesis, which allows plants to absorb energy from light for carbon dioxide fixation. The most green leaf material contains about 0.3% chlorophyll and on extraction using conventional methods yields about only 5%. Cheap sources, like grass and lucerne (alfalfa), are usually selected as chlorophyll source [35]. In more esoteric extractions, leaves of nettles and elder are preferred, whilst algae and silk worm droppings have also been used commercially [36].

Structurally, chlorophyll is essentially two parts: a substituted porphyrin ring known as an excellent chelating ligand and a long carbon chain phytol [37]. Dietary chlorophyll is predominantly composed of lipophilic derivatives including chlorophyll a and b. The structural modifications of the chlorophyll pigments mainly the de-esterification of phytol ring may significantly increase (65-fold) its transfer from the food matrix to the intestinal epithelial cells during digestion [38]. Sodium/copper derivatives of chlorophyll are frequently used as food additives or in beverages [39]. Sodium copper chlorophyllin is an approved food color in the USA (http://www.accessdata.fda.gov/scripts/cdrh/cfdocs/cfCFR/CFRSearch.cfm?fr=73.125). Besides that, it repairs cells, increases hemoglobin in blood and promotes the cell growth [5]. Regarding the cytotoxity of chlorophyll derivatives, the application of pheophorbide and pheophytin against tumor cell revealed that the cellular uptake and inhibition of myeloma cell multiplicity was greater for pheophorbide than pheophytin, hence it indicates that chlorophyll derivates may play a role in cancer prevention [28]. Patients with chronic relapsing pancreatitis can be treated with chlorophyll-α without any unfavorable side-effects, such as of allergic, or photosensitive, or hepatotoxic nature [40].

Chlorophyll has also been investigated as potential source of pigments in cosmetics; the brown and red algae are mostly used in the cosmetic industries [5]. Furthermore, in food industry, chlorophyll is used as natural pigment ingredient in processed foods. Because of its strong green pigment and consumer demand for natural foods, chlorophyll is gaining importance as food additive. This in turn is encouraging food processors to switch from artificial pigments to chlorophyll-based natural coloring. However, a downstream process needs to be developed to purify chlorophyll a and b from algae [5].

### Phycobiliproteins

Phycobiliprotein, a high-potential molecule, has been utilized commercially as natural dyes and has a variety of applications in pharmaceutical industry. It is classified according to UV-visible absorption maxima as phycocyanin (blue pigment), phycoerythrins (red pigment), and allophycocyanin (pale-blue pigment). The annual market of phycocyanin was around US$5-10 million [41]. The Cyanobacterium *Spirulina* sp. is a source of c-phycocyanins and allophycocyanins and contains relatively low nucleic acid content; is composed of 55%-70% protein, 6%-9% fat, and 15%-20% carbohydrate, and is rich in minerals, vitamins, fibres, and pigments [42]. Another type of algae that can be a source for phycobiliprotein is *Aphanizomenon flos*-*aquae*[28, 43]. *Arthrospira* sp. is a group of Cyanobacteria characterized by loosely spiral shaped trichomes arranged in an open helix enclosed in a thin mucilaginous sheath, usually found in alkaline, brackish and saline waters. They usually become predominant species and form massive blooms [44]. The phycobiliprotein may be utilized for labeling antibodies in clinical tests (such as immunofluoresscence or flow cytometry) by binding to biologically active molecules such as immunoglobulin, biotin or protein [28, 45]. Scientific studies have shown that the synergistic action of a wide spectrum of antioxidants is better than the activity of a single antioxidant, and that antioxidants from natural sources (primarily foods) have a higher bioavailability and therefore higher protective efficacy than synthetic antioxidants [43]. It is reported that phycocyanin acts as antioxidant against free radicals and selenium and phycocyanin have been reported to show potent cancer chemopreventive activities [46, 47]. It is proven that phycocyanin is 20 times more efficient than ascorbic acid against hemolysis induced by peroxyl radicals in human erythrocytes [28].

### Polyunsaturated fatty acids (PUFAs)

Polyunsaturated Fatty Acids (PUFAs) include docosahexaenoic acid (DHA), eicosapentaenoic acid (EPA), arachidonic acid (AA), γ-linolenic acid (GLA) and have been widely recognized as beneficial towards human health [48]. According to a new technical market research report “Global Market for Oleochemical Fatty Acids” (CHM062A) from BCC Research (http://www.bccresearch.com), the naturally produced fatty acid market was valued at US$7.2 billion in 2011 and should reach US$6.9 billion in 2012. The total market value of fatty acids expected to reach US$13 billion in 2017 (http://www.slideshare.net/bccresearch).

Natural PUFAs can be obtained from fish or extracted from fish oil. However, there is report on the possibility of accumulated toxin in the PUFAs derived from fish sources, furthermore the unpleasant smell, taste and poor oxidative stability has limits the application of fish oil as food additive [4]. Fish oil derived PUFAs have low oxidative stability and the oxidative stability of DHA containing algae oils varies widely depending on some physical parameters. However, EDTA can be used to inhibit oxidation of foods containing long-chain PUFA from either fish or algae and fortified with iron [49]. Moreover, a recent study has shown that PUFAs (450 mg day^-1^) are not being met by the diet in the majority of the population; the current mean intake of PUFAs by adults is estimated to be about 282 mg day^-1^ (EPA and DHA contributing about 244 mg day^-1^) [48]. It is undeniable that microalgae can be the alternative natural source for PUFAs; PUFAs found in fish are actually originated from microalgae. However, DHA is the only algal PUFA commercially available as an omega-3 fatty acid and has been documented as a major structural fatty acid in the grey matter of the brain [50]. DHA is important for correct brain and eye development in infants and has been shown to support cardiovascular health in adults [4, 51]. DHA helps body to fight against diseases and recent studies explore the significant role of PUFAs in the immune system through peroxisome proliferator-activated receptors (PPAR-α and PPAR-γ), and favorable effects of n-3 PUFA supplementation on allergic diseases and novel therapeutic strategies to treat eosinophilic disorders have also been reported [52].

Μarine microalgae have significantly higher DHA contents compared to fresh water microalgae [5]. DHA is found in a limited selection of foods such as fatty fish and organic meat; it also occurs naturally in breast milk and its concentration in breast milk might be influenced by environmental factors, however it is not available in cow’s milk [53]. Since 1990, a number of health and nutrition organizations have specifically recommended the inclusion of DHA in infant formula for preterm and full term infants. DHA is the characteristic PUFA of the marine dinoflagellates. *Crypthecodinium cohnii* is a non-photosynthetic, marine dinoflagellate producing DHA predominantly [54]. Nearly 30-50% of its constituent fatty acids are C22:6 (n-3) fatty acid and no other PUFAs are present in excess of 1%. It is, thus easy to separate DHA from the fatty acid mixtures. For this reason, *Crypthecodinium cohnii* represents a promising source for the commercial production of DHA [55]. The world wholesale market for infant formula was estimated to be about US$10 billion per year [56]. A number of food items containing PUFAs (including DHA) are being sold in market [4]. Some algae-related heterotroph strains that have high DHA such as *Schizochytrium mangrove*, reported to have main component of DHA in a range of 33-39% of total fatty acid while *Amphidium caryerea* and *Thrautocytrium aureum* contain 17% and 16.1%, respectively [5].

*Nannochloropsis sp*. has been proposed as a source of PUFAs due to its high contents of EPA [57]. Thus, *Nannochloropsis sp*. production has been used to address the rotifer requirements of 8 million gilt-head sea bream fingerlings production annually [58]. Being suitable for aquaculture industry, EPA too has been used as nutritional supplement. The diatoms *Phaeodactylum tricornutum* and *Nitzschia laevis* are algae strains to be utilized as sources of EPA [4, 59]. *Chaetoceros muelleri* var. *subsalsum* and *Isochrysis galbana* have record percentage of DHA and EPA; they present the highest output of EPA (3.5% and 4.8% on dry basis, respectively) using vertical plate glass reactor [58, 60]. EPA is an n-3 PUFA that plays an important role in the regulation of biological functions and prevention and treatment of a number of human diseases such as heart and inflammatory diseases [61]. The eicosanoids are hormone-like substances including prostaglandins (PG), thromboxanes (TX) and leukotrienes (LT). AA and EPA are precursors of eicosanoid compounds. However, the eicosanoids from these two fatty acids are different structurally and functionally, and are sometimes even antagonistic in their effects. A balanced uptake of EPA/AA can prevent eicosanoid dysfunctions and may be effective in treating a number of illnesses and metabolic disorder [59]. *Porphyridium cruentum*, a red alga, has been used for massive cultures due to its relatively high AA and EPA concentration. Nevertheless, *Porphyridium cruentum* biomass is high in protein (34.1%), carbohydrates (32.1%) and essential minerals [62]. AA is potentially used for infant formulas (either for full-term or preterm infants) and as nutritional supplement [4].

Aside from phycobiliprotein, *Arthrospira* sp. is known to be the richest algal source of GLA. GLA is important to the infant formulas for full-term infant and has the potential to lower low-density lipoproteins in hypercholesterolemic patients, alleviate the premenstrual syndrome and treat atopical eczema [4, 44]. GLA may attenuate the signs and symptoms of inflammatory diseases such as rheumatoid arthritis and atopic dermatitis [63, 64]. From both animal and human trials, it has been revealed that dietary supplementation with GLA can modulate inflammation [63].

### Beta-1,3-glucan

Beta-1,3-glucanis considered responsible to initiate host defense reactions in response to pathogen surface molecules. The most important substance other than oil in *Chlorella* sp. is beta-1,3-glucan. The world annual sales of *Chlorella* sp. are in excess of US$ 38 billion [65]. Initially, beta-1,3-glucan was named as Zymosan in the 1940s in Norway. It was found in the cell wall of baker’s yeast. Further studies have been conducted by Riggi & Di Luuzio in 1961 [66] in Italy, and since then the bio-active compound was renamed as beta-1,3-glucan. Further studies on beta-1,3-glucan has been done in Japan around 1970s. Beta-1,3-glucan is important to human health. Feeding of immunostimulant beta-1,3-glucan to healthy fish may raise the non-specific and specific immunity level and the protection against bacterial infection compared with the control [67]. *Chlorella* sp. is a major source of beta-1,3-glucan [68]. Beta-1,3-glucan had significant effect as a prophylactic treatment to reduce the anthrax infection in mice and the same type of treatment can also inhibit the growth of cancer cells *in vivo* by involving stimulation of three important cytokines [69, 70]. Positive effects were also found in patients after cardiopulmonary bypass, and inhibition of antiviral activity has been found in HIV-infected patients, moreover beta-1,3-glucans are routinely used in patients for tumor immunotherapy [71]. The use of beta-1,3-glucan is of special interest in the cancer patient undergoing chemotherapy and/or radiation treatment, since beta-glucans have shown a remarkable ability to accelerate hematopoetic recovery in both sublethally and lethally irradiated mice. It can also stimulate recovery of the bone marrow following chemotherapy, which is vital to restricting tumor growth and preventing infectious complications during treatment. Amparyup *et al*. [72] showed that pattern recognition proteins [including lipopolysaccharide and beta-1,3-glucan binding protein (LGBP)] could enhance the phenoloxidase activity in shrimp.

### Pharmaceutical and nutraceutical compounds

Algae have the potential to be a source of compounds having antibiotic and anticancer activity. A wide variety of Cyanobacteria produce compounds including sulfolipids that were active against herpes virus, pneumonia virus and HIV [73, 74]. Cyanobacteria can produce tolytoxin (6-hydroxy-7-O-methylscytophycin B), an anti-fungal antibiotic and highly toxic to mice [75, 76]. The marine cyanobacterium *Lyngbya majuscula* produces aurilides B and C having cytotoxicity against human lung tumor and mouse neuroblastoma cell line [77]. Moreover the macroalgae *Laphocladia* sp. is capable of producing lophocladine alkaloids that exhibit cytotoxicity to NCI-H460 human lung tumor and MDA-MB-435 breast cancer cell lines [78]. Therefore, once the algal sources of useful medicinal compound are identified, the algae can be cultivated in mass scale and the material can be extracted, purified, and marketed [1]. Some microalgae species are established in the skin care market, the main ones being *Arthrospira* sp. and *Chlorella* sp. A number of cosmeticians have even invested in their own microalgae production (http://www.fao.org/docrep/012/i1704e/i1704e01.pdf). Two examples of commercially available products and their properties claimed by their companies are a) a protein-rich extract from *Arthrospira* sp. that repairs the signs of early skin aging, exerts a tightening effect and prevents stria formation [4] (Protulines, Exsymol S.A.M., Monaco) and b) an extract from *Chlorella vulgaris* that stimulates collagen synthesis in skin, thereby supporting tissue regeneration and wrinkle reduction (Dermochlorella, Codif, St. Malo, France http://www.coptis.com/site/desc.php?RefFab=1CDF). Recently, two new products have been launched by Pentapharm (Basel, Switzerland): a compound from *Nannochloropsis oculata* is used for excellent skin-tightening properties (short and long-term effects) (Pepha-Tight) and an ingredient from *D. salina* is known to have the ability to markedly stimulate cell proliferation and turnover to positively influence the energy metabolism of skin (Pepha-Ctive) [4].

### Livestock feed

Spirulina has been frequently used in feed supplements due to its excellent nutrient compounds and digestibility because it has small amount of carbohydrates. The livestock feed is another useful product which may be obtained from the algae, since many of algae species have been examined for their biochemical compositions to evaluate their suitability as a substitute or primary livestock feed [79]. Spirulina has a unique blend of nutrients containing nutrients that include B-complex vitamins, minerals, proteins, gamma-linolenic acid and super anti-oxidants (such as beta-carotene, vitamin E, trace elements) and a number of unexplored bioactive compounds [80]. Compared to other microorganisms, *Spirulina* sp. can be cultivated in high saline water and alkaline conditions which give an advantage to function as a feedstock for livestock feed [81]. Recently, studies regarding growth and body conformation responses of genetically divergent Australian sheep to Spirulina (*Arthrospira platensis*) supplementation revealed that Spirulina can increase live weight, growth and body conformation significantly [82]. Feeding lipid-encapsulated algae supplements may increase n-3 content in milk fat without adversely affecting milk fat yield [83].

An algal supplementation level of about 10 g kg^-1^ of dry matter intake proved to be effective in reducing milk fat content and in modifying the milk fatty acid composition toward increased conjugated linolenic acid (CLA) and DEA concentrations [84]. A related study demonstrated that dietary supplementation with fish meal or n-3 PUFAs in early-lactating dairy cows significantly increased milk yield with no change in milk composition [85]. In addition, some sea weeds like red algae (e.g. *Porphyra* sp., *Gracilaria* sp. and *Kappaphycus* sp.) and brown algae (particularly *Laminaria* sp., *Undaria* sp. and *Hizikia fusiforme*) were used in human food exhibiting potential to be used for livestock feed. *Porphyra* sp., *Gracilaria* sp., *Grateloupia* sp., and *Kappaphycus* sp. have also been genetically transformed for enhanced production of respective products [86]. The annual *Porphyra* sp. harvest worldwide has been estimated to be worth of US$2.5 billion. *Porphyra* sp. primarily contains vitamin A, vitamin B, vitamin C, beta-carotene, as well as essential minerals including iodine. Besides that, a study on *Laminaria digitata* suggested that algae supplemented feed has increased the pig weight up to 10% on a daily basis [1, 5].

### Poultry feed

There are more chickens in the world than any other bird; in fact, more than 50 billion chickens are reared annually as a source of food, for both their meat and eggs (http://www.ciwf.org.uk/farm_animals/poultry/). Comprehensive analyses and nutritional studies have demonstrated that algae contain proteins and these proteins are of high quality and comparable to conventional vegetable proteins [87]. Ginzberg *et al*. [88] studied the role of algae *Porphyridium* sp. as feed supplement on metabolism of chicken. It was found that cholesterol of egg yolk was reduced about 10% and color of egg yolk became darker, indicating the production of higher carotenoids [5, 89]. In poultry rations, algae up to a level of 5-10% can be used safely as partial replacement for conventional proteins. Prolonged feeding of algae at higher concentrations may cause adverse effects. The yellow color of broiler skin and shanks as well as of egg yolk is the most important characteristic that can be influenced by feeding algae. Moreover, the Institute für Getreideverarbeitung (Bergholz-Rehbrücke, Germany) produces a natural feed with the algae *Chlorella* sp. and *Arthrospira* sp. called Algrow [90].

### Aquaculture feed

Demand for fish for human consumption has risen due to the environmental concerns over Open Ocean fishing [91]. The world market for farmed fish and aquatic edible plants was valued in 2008 at US$106 billion. Products from fisheries and aquaculture combined are supplying the world with 142 million tonnes of protein every year (http://www.blueeconomy.eu/m/articles/view/Fish-without-Feed-Part-1-The-Market).

According to another report by United Nations Food and Agriculture Organization (http://www.fao.org/docrep/016/i2727e/i2727e.pdf), in the last three decades (1980-2010), world food fish production of aquaculture has increased by almost 12 times, at an average annual rate of 8.8% and the world aquaculture production attained another all-time high in 2010 at 60 million tonnes (excluding aquatic plants and non-food products), with an estimated total value of US$119 billion (http://aquabounty.com/aquaculture-market/). Non-toxic marine microalgae, including the stramenopiles *Isochrysis* sp., *Pavlova* sp. and *Nannochloropsis* sp. as well as various diatoms, represent the primary food source at some stages in the life cycle of most cultivated marine animals [1]. Therefore, algal biomass will be in high demand for the fish-food and aquaculture markets in the future and provide ample revenues for the algae industry [91].

A challenge in fish nutrition is to generate end products with high levels of health-promoting long chains of omega-3 fatty acids for the consumer, while reducing the use of fish oils. Omega-3 oils have a high market demand in both the human nutraceuticals and animal feed industries. This growing trend is an additional driving force for the marketing of non-marine-based omega-3 oils and alternative feed ingredients. Algae is a rich source of high quality protein, vitamins, micronutrients (trace elements), and carotenoids, Long-chain PUFAs, especially of n-3 and n-6 series such as eicosapentaenoic (EPA), docosahexaenoic (DHA), and arachidonic (AA) are considered pharmacologically important for dietetics and therapeutics [90]. Algal meal is a rich source of high-quality protein, vitamins, micronutrients (trace elements) and carotenoids, which can be used directly in aqua feeds [85]. Furthermore, fish phosphoglycerides generally contain 50% of their total fatty acids as n-3 PUFA with a ratio of 22:6 (n-3):20:5 (n-3) of about 2:1 and it can be seen most clearly in the phosphoglycerides of fish eggs [59]. In addition, better growth weight and protein efficiencies ratio of tilapia was observed when supplied with algae as nutritional source in feed. Also, *Phorphidium valderianum* was successfully used as feed for aquaculture (based on its nutritional performance and non-toxic properties) [5]. The diatom *Thalassiosira pseudonana* is widely cultivated to feed variety of mollusks, including the Pacific oyster *Crassostrea gigas* and rock scallops [1]. The long chain PUFAs are highly susceptible to oxidation because of their unsaturated nature; the addition of natural antioxidants to the highly unsaturated fish oil may protect it from oxidation [92].

### Shrimp feed

The total global production of farmed shrimp reached more than 1.6 million tonnes in 2003, representing a value of nearly US$9,000 million (http://fisherymanagement.wikia.com/wiki/Shrimp_farm). Shrimp farming production reached 737,200 tonnes in 1998, an increase of 12% from 1997. This increased production was mainly reported in subtropical regions of America (28%, 457 hatcheries) and SE Asia (72%, 3,718 hatcheries) [59]. According to Shrimp Production Review by Food and Resource Economics Department, University of Florida USA, the shrimp farming was estimated to be about 4 million metric tonnes in 2013, with 10.3% projected annual growth rate (mainly attributed to SE Asian countries and China) (http://www.gaalliance.org/update/GOAL11/DiegoValderrama.pdf). However, disease outbreaks in recent years have affected farmed Atlantic salmon in Chile, oysters in Europe, and marine shrimp farming in several countries in Asia, South America and Africa, resulting in partial or sometimes total loss of production (http://www.fao.org/docrep/016/i2727e/i2727e.pdf). In 2010, aquaculture in China suffered production losses of 1.7 million tons caused by natural disasters, diseases and pollution. Disease outbreaks virtually wiped out marine shrimp farming production in Mozambique in 2011 (http://www.fao.org/docrep/016/i2727e/i2727e.pdf). Microalgae species *Hypnea cervicornis* and *Cryptonemia crenulata* particularly rich in protein were tested in shrimp diets. Amount of algae in fish meal resulted in significant increase in shrimp growth rates [5]. The requirement for DHA in marine fish and shrimp nutrition has been established via feeding diets containing DHA and AA; the findings revealed that shrimp rose on diets rich in DHA and AA showed significant improvement in immune parameters, such as total hemocyte count, phenoloxidase activity, superoxide dismutase activity, and bactericidal activity [93]. Recently, it has been reported that the addition of 0.5% Spirulina meal in a complete diet for shrimp (*L. vannamei* juveniles), with 14% of Peruvian fishmeal, has proved as a nutritionally efficient feeding attractant [94]. Through short-term feeding studies, two marine algal products (MAP) named MAP3 and MAP8 were found suitable as substitute of protein source in the feeds of three prominent farmed species [89]. The studies regarding the utilization of fish oil and algae as a source of PUFAs are going side by side but the trends are more inclined towards algal products. Menhaden fish oil can also be used as a source of dietary fatty acids which influence the composition of Pacific white shrimp muscle tissue by exhibiting significantly higher shrimp muscle DHA level [95]. As fish meal content, DHA is required for the production of ecdysone for molting, growth and egg production. The mechanism involves the biological membranes rich in di-22:6 (n-3) phosphoglycerides which is relatively constant in the face of changing environmental variables like temperature, pressure, and salinity [59].

## Conclusion

Microalgae have multiple potential to produce high-valued products and there is an urgent need of awareness makes these biomolecules popular in the world to meet the increasing demands with respect to population. Most of these biomolecules are not produced in the animal/human body but termed as essential; therefore, it is highly recommended to make these biomolecules available for food and feed purposes. Although the production of biomolecules from algae requires some awareness regarding technology and market value at the industrial and farmer level, it has been proved that these biomolecules can be produced using algae as a potential source at small or large scale. The demanding market of these biomolecules is quite convincing to stimulate research interests leading to production programs by the related organizations. Algae culturing got attention because of its biofuel producing characteristics but the awareness regarding cultivation technologies may also lead to set trends for biomolecule production as a pharmaceutical or nutritional product. The production of biomolecules from algae and their subsequent utilization in animal and aquaculture feed may support the objectives to establish industry from microalgae at domestic or industrial scale. Some of the biomolecules can be produced as a byproduct while producing biofuel from algae as a main targeted product. Eighty-two percent of the world fish stocks are overexploited, depleted or endangered, while demand for fish protein is exploding. With the world population expected to reach 9 billion by 2050, demand for critical sources of protein continues to outstrip supply.

## Consent

Written informed consent was obtained from the patient for the publication of this report and any accompanying images.

## Abbreviations

AA: Arachidonic acid
CLA: Conjugated linolenic acid
DEA: Drug Enforcement agency
DHA: Docosahexaenoic acid
DNA: Deoxyribonucleic acid
EDTA: Ethylene diamine tetra acetic acid
EPA: Eicosapentaenoic acid
GLA: (Gamma) γ-linolenic acid
HIV: Human immunodeficiency virus
LGBP: Lipopolysaccharide and beta-1,3-glucan binding protei
LT: Leukotrienes
M: Molar
MAP: Marine algal products
MGO: Marigold Orange
PG: Prostaglandins
PPAR: Peroxisome Proliferator-activated receptors
SE: South East
Sp: Specie
TX: Thromboxanes
UV: Ultraviolet
UVA: Ultraviolet A.
